# Vitamin Nutritional Status in Patients with Pancreatic Cancer: A Narrative Review

**DOI:** 10.3390/ijms25094773

**Published:** 2024-04-27

**Authors:** Elena Azzini, Tiziano Furini, Angela Polito, Luca Scalfi, Alessandro Pinto, Valeria Gasperi, Isabella Savini, on behalf of the SINU “Nutrition in Oncology” Working Group

**Affiliations:** 1 Council for Agricultural Research and Economics—Research Centre for Food and Nutrition, 00178 Rome, Italy; angela.polito@crea.gov.it; 2 Department of Experimental Medicine, University of Rome “Tor Vergata”, 00133 Rome, Italy; nutrizionefurini@gmail.com (T.F.); gasperi@med.uniroma2.it (V.G.); 3 Department of Public Health, School of Medicine, Federico II University, 80131 Naples, Italy; scalfi@unina.it; 4 Experimental Medicine Department, Food Science and Human Nutrition Research Unit, “Sapienza” University, 00185 Rome, Italy; alessandro.pinto@uniroma1.it

**Keywords:** pancreatic cancer, water-soluble vitamins, fat-soluble vitamins, vitamin nutritional status

## Abstract

Due to the high mortality rate in Western countries, pancreatic cancer is considered one of the *big killers*, leaving patients and their families with little hope upon diagnosis. Although surgical and drug therapies are critical for cancer patients to improve life expectancy and alleviation of suffering, nutrition plays a key role in improving cancer treatment outcomes. This narrative review, conducted as part of the activities of the Italian Society of Human Nutrition (SINU) working group in oncology, focuses on the prevalence of vitamin malnutrition among pancreatic cancer patients. The results of the literature search show that pancreatic cancer patients are at a heightened risk of water-soluble vitamin deficiencies, particularly of vitamins B1, B3, and B6. Additionally, they also face an increased risk of deficiency of fat-soluble vitamins. Among these vitamins, the potential role of vitamin D in pancreatic cancer has garnered the most attention, with its plasma levels being identified as a significant factor in patient survival. Investigating vitamin nutritional status could provide valuable insights for incorporating nutritional approaches into the prevention and treatment of pancreatic cancer, thereby reducing the exacerbation of symptoms associated with the diagnosis.

## 1. Introduction

In recent decades, the interest in the role of nutrition has become increasingly relevant, especially in the context of noncommunicable diseases (NCDs), including cancer. Although for cancer patients the main aspects concern surgical and pharmacological therapies, life expectancy, and alleviating suffering, nutrition plays a fundamental role in improving outcomes of oncological treatments. In fact, malnutrition is often underestimated and even if diagnosed, is untreated in 50% of cases [1]. Due to treatments and living conditions, cancer patients have metabolic alterations, leading to changes in body composition, including sarcopenia, cachexia, or sarcopenic obesity, and/or nutritional deficiencies. It has been estimated that up to 51% of cancer patients have a nutritional deficiency, 9% are overtly malnourished, and 43% at risk for malnutrition; the severity of malnutrition is positively correlated with cancer stage [2]. As a result, malnutrition associated with cancer can cause various impairments, impair physical function, and decrease survival. There are cases of micronutrient deficiencies that can lead to worsening of symptoms; particularly, vitamin deficiencies can cause further deterioration in the patient’s quality of life. In this context, the lack of information about the micronutrient status in patients with pancreatic malignancies often referred for surgery should be highlighted, as micronutrient deficiency states could impact their recovery. Hence, an evaluation of specific vitamins aimed at highlighting borderline status in these patients may be useful [3]. According to the Italian Ministry of Health documents, the goal of screening in a cancer patient nutritional pathway is to: (i) address nutrition from the diagnosis of cancer pathology; (ii) optimize and consolidate the effects of cancer therapy; (iii) limit the side effects; (iv) improve the quality of life; (iv) prevent metabolic complications of cancer and chemotherapeutic therapies [4]. This narrative review, conducted as part of the activities of the Italian Society of Human Nutrition (SINU) working group in oncology, focuses on the prevalence of vitamin malnutrition among pancreatic cancer (PaC) patients. PaC is considered one of the big killers (i.e., tumors with the highest mortality rate in Western countries), leaving patients and their families with little hope upon diagnosis. Although surgical and drug therapies are critical for cancer patients to improve life expectancy and alleviation of suffering, nutrition plays a key role in improving cancer treatment outcomes, and its diagnosis often leaves little hope. Estimates of the temporal trends of PaC incidence and mortality produced by GLOBOCAN 2020 indicate increases in both incidence (with 495,773 new cases, 2.6% of all sites) and mortality (with 466,003 new deaths, 4.7% of all sites) [5]. Besides hormones, pancreas produces digestive enzymes, which play an important role in vitamin absorption and utilization; consequently, cancerous pancreatic tissues lead to additional complications, further exacerbating patient symptoms already impaired by cancer. As tumor development and progression is multifactorial, where genetic factors, family history, and lifestyle play crucial roles [6], the study of nutritional aspects (in terms of macro- and micronutrient status) of the disease might allow for considering a nutritional approach as part of the prevention and/or therapy of PaC.

Against this background, the present review aims at providing an overview on vitamin status in PaC patients.

## 2. Results

It should be noted that vitamin deficiencies are poorly studied overall. The body’s vitamin requirements are small doses, in the milligram or microgram range, and can generally be met by a varied diet that does not exclude any type of food and includes the regular consumption of vegetables and fresh fruit. Deficiencies are, therefore, rare and typically caused by inadequate intake of vitamins due to an unbalanced diet or failure to adapt to conditions that increase their requirements (such as pregnancy, lactation, or growth). Additionally, they can be caused by diseases that impair their absorption and metabolism, such as digestive system diseases, cancer, and alcoholism. Although few studies exist on vitamin deficiencies in PaC patients, it is still possible to gather data on both water-soluble and fat-soluble vitamins. Possible vitamin deficiencies may be caused by anorexia, symptoms caused by oncologic treatment and surgery, and the patient’s quality of life. The limited number of clinical trials aimed at improving the understanding of adequate nutritional support in different care settings for cancer patients, unstructured collaboration between oncologists and clinical nutrition specialists, and persistent insufficient awareness of nutritional issues among health care providers may further contribute to the impact of these micronutrient deficiencies [7].

Studies that consider the effects of vitamins in patients with PaC generally do not report basal concentrations in patients with cancer compared to controls or references, nor nutritional screenings or correlations with other variables (e.g., underweight, malnutrition, and body composition). The paragraphs that follow contain information on each vitamin in relation to its main characteristics and nutritional status in PaC patients.

### 2.1. Water-Soluble Vitamins

**Figure 1:**
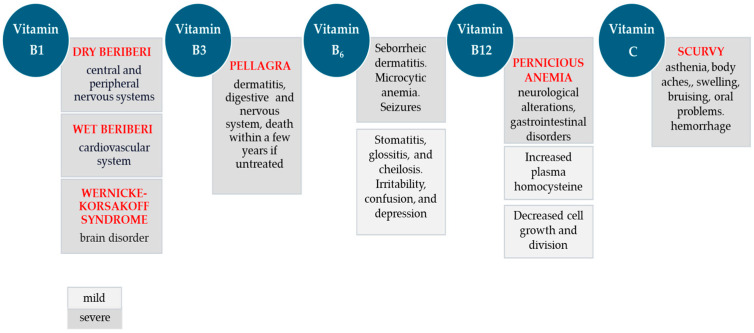
Some consequences of water-soluble vitamin deficiencies.

Water-soluble vitamins include the vitamin B complex and vitamin C and generally exert coenzyme activity. This means that they activate and cooperate with enzymes to facilitate biochemical reactions. In essence, they act as biocatalysts. Due to their solubility in water, these vitamins cannot be stored in the body to form reserves. Therefore, even after short periods of inadequate intake, conditions of malabsorption or metabolic changes that increase their requirements, changes occur in various metabolic pathways, with consequent damage to cells, tissues, and organs. In the early stages of mild deficiency, clinical signs are difficult to detect because the symptoms are not specific. As shown in Figure 1, in later stages, specific vitamin deficiencies become severe and are associated with well-defined diseases, such as beriberi or scurvy.

In PaC patients, the highest risk of water-soluble vitamin deficiency involves thiamine (vitamin B1), niacin (vitamin B3), and pyridoxine (vitamin B6). Regarding the other water-soluble vitamins, there is relatively little evidence and it often concerns studies that consider neoplasms of multiple organs, thus resulting in less focus on the pancreas.

Data about folates (vitamin B9) are scarce and unclear. In their patients, Tabriz et al. found plasma levels within the reference range in the follow-up because of pylorus preserving duodenocephalopancreasectomy [8]. For riboflavin (B2), only recent findings from a Chinese prospective cohort study [9] suggest a potential role of riboflavin in the development of PaC, especially in men. However, this search did not provide any results for pantothenic acid (vitamin B5).

#### 2.1.1. Vitamin B1

Thiamine, whose active form is thiamine pyrophosphate (TPP), is a coenzyme participating in the metabolism of carbohydrates and branched-chain amino acids and is involved in energy production [10]. Food sources of vitamin B1 have both animal and plant origin (high concentrations are found in whole grains and legumes) [11]. Thiamin deficiency mainly affects cardiovascular and central and peripheral nervous systems (causing different types of beriberi and Wernicke–Korsakoff syndrome) [10]. Due to its role in regulating energy metabolism, thiamin deficit can also impact other cells and tissues. Therefore, a protective role of thiamin against PaC has been hypothesized, although available data on molecular mechanisms derived from in vitro studies are scarce and conflicting [12]. The evaluation of thiamine levels by measuring erythrocyte transketolase activity up to 15% is indicative of acceptable nutritional status. An increase of 16–24% indicates a mild deficiency, while an increase of >25% indicates a deficiency. Urinary excretion may also be considered [13]. The average thiamine storage capacity is approximately 18 days, and its deficiency can occur in any condition of malnutrition [14]. It has been postulated that the thiamine deficiency observed in abdominal cancer patients may be caused by nutrient malabsorption because of the impact on digestive enzyme production and/or lack of appetite, also caused by chemotherapy [15,16]. A case study of a woman with PaC treated with paclitaxel and gemcitabine-based chemotherapy for 6 months showed that the patient presented loss of appetite and, consequently, thiamine deficiency [14]. In addition to appetite loss, an additional cause of thiamine deficiency (and of other micronutrients, such as vitamins B6, E, and D and iron) may be duodenocephalopancreasectomy surgery with pylorus preservation. In patients undergoing this surgical treatment, thiamine levels have been found lower than normal reference values. Tabriz et al. reported attainment of physiological plasma levels only 12 months after surgery [8]. Several case studies seem to indicate that after the Whipple procedure, vitamin B1 deficiency can early lead to Wernicke’s encephalopathy [17] and its supplementation is necessary to restore normal cognitive functions and prevent permanent brain damage [18]. As reported in a recent case study, Wernicke’s encephalopathy is an uncommon condition due to thiamine deficiency and is very rarely associated with acute pancreatitis, occurring late in the PaC course [19]. Preliminary studies suggest that thiamine deficiency may also be caused by fluorouracil-based chemotherapy [20], which is one of the most used chemotherapies for the treatment of PaC, even though these data still need to be confirmed. Furthermore, the preoperative nutrition status in pancreatoduodenectomy patients was reported to be significantly correlated with their prognosis [18].

#### 2.1.2. Vitamin B3

Vitamin B3, with the term niacin—also called PP (pellagra-preventing)—refers to two vitamers, nicotinic acid and nicotinamide, giving rise to two oxidoreductive coenzymes, nicotinamide adenine dinucleotide (NAD) and nicotinamide adenine dinucleotide phosphate (NADP). Both coenzymes participate in redox reactions fundamental for energy production [21]. Humans obtain niacin from both endogenous (from 2% of dietary tryptophan) and exogenous (animal and vegetable foods) sources [21]. Niacin nutritional assessment is inferred from the metabolite N-methyl nicotinamide detected in urine or from the erythrocyte NAD/NADP ratio [11]. NAD also acts as a substrate of sirtuin 1 (SIRT1) and poly ADP-ribose polymerase (PARP1), two important non-redox enzymes involved in deacetylation reactions and poly ADP-ribosylation, respectively. Consequently, niacin crucially impacts gene expression, DNA repair, apoptosis, and cell proliferation [22,23]. In line with this property, a protective role has been postulated for niacin against different cancer types, even if only preclinical studies have investigated its efficacy against PaC [24,25]. Concerning niacin status in PaC, its deficiency was observed in patients with gastroenteropancreatic (GEP) neuroendocrine tumors (NET), especially in those producing serotonin [26], where tryptophan deficit leads to a subclinical niacin deficiency (with a prevalence of around 45%). Shah et al. [27] found significantly lower serum niacin levels in about 28% of NET patients with carcinoid syndrome (CS) with respect to the controls (13%). Clement and coworkers reported that about 5% of patients with CS can develop pellagra (with symptoms of dermatitis, diarrhea, and dementia), as result of tryptophan deficiency [28]. Laing and colleagues highlighted that niacin deficiency and risk of pellagra increase over time and are potentially more prevalent in patients with a prolonged history of advanced NET [29]. Moreover, up to 80% of patients with CS, due to the advanced stage of their NET disease, died after pellagra identification [30].

#### 2.1.3. Vitamin B6

Vitamin B6 refers to three different vitamers derived from 2-methyl-3-hydroxy-5-hydroxymethylpyridine: pyridoxine, pyridoxal, and pyridoxamine. The coenzyme forms are pyridoxamine-5′-phosphate (PMP) and pyridoxal-5′-phosphate (PLP). Vitamin B6 is found in foods such as meat, fish, offal, whole grains, legumes, and vegetables. PLP and PMP are the metabolically active forms and are estimated to be essential for 4% of all enzyme activities in the human genome [31]. The bioactivity of vitamin B6 and its vitamers has been demonstrated in in vitro tumor models through the inhibition of cell proliferation, the enhancement of cytotoxicity of chemotherapy, and alterations in gene expression. Recently, He et al. [32] suggested the inhibition of tumor growth in PDAC models through vitamin B6 supplementation with the blockade of VB6-dependent one-carbon metabolism and amplification of the anti-tumor immunity of NK cells. Their results emphasize the importance of nutritional competition among different components of the tumor microenvironment in determining tumor growth, immune tolerance, and therapeutic resistance. Vitamin B6 nutritional status can be assessed mainly by plasma PLP concentration and by urinary excretion of catabolites of the vitamin or tryptophan and erythrocyte glutamate-oxaloacetate transaminase activity [33]. Evidence on B6 deficiency was found in a study of patients undergoing duodenocephalopancreasectomy with pylorus preservation. Tabriz et al. [8] found that vitamin B6 levels were lower at all stages of treatment, both pre- and post-surgery. A case study of a 31-year-old woman [34] revealed the presence of PaC causing acrodermatitis enteropathic-like eruption associated with decreased pyridoxine absorption (as well as cobalamin and vitamins A and C). A recent meta-analysis that included nine studies tracking participant vitamin B6 intake and the development of PaC suggested that vitamin B6 intake significantly decreases PaC risk [35]. A similar finding has also been reported by another metanalysis [36]. Even though this evidence stems from case–control studies, the principal circulating form of vitamin B6 (PLP) is associated with a significant linear decreased risk of PaC. However, further studies are needed to clarify and confirm the role of vitamin B6 in this cancer.

#### 2.1.4. Vitamin B12

Vitamin B12 serves as a cofactor for folate-dependent methionine synthesis, thus playing an essential role in folate metabolism. Besides acting as a coenzyme in the synthesis of nucleic acids, it is involved in the formation of red blood cells and myelin [37]. The main food sources of this vitamin (also known as cobalamin) are those of animal origin, while plant foods (except for some seaweeds) are almost completely devoid of it. Intrinsic factor, a protein synthesized in the stomach, is required for the absorption of this vitamin in the intestine, and consequently, some forms of gastritis result in vitamin B12 deficiency, which manifests as a severe form of anemia (pernicious anemia). Concerning vitamin B12 in PaC patients, there are few and conflicting literature data: patients undergoing pylorus preserving duodenocephalopancreasectomy appear to have no vitamin B12 deficiency [8], while a case study indicates that patients with NET may be experiencing cobalamin deficiency [34]. Additional studies, therefore, are needed to clarify the relationship between vitamin B12 and PaC.

#### 2.1.5. Vitamin C

As is well known, vitamin C (ascorbic acid) is an antioxidant that can act at higher pharmacological doses as a pro-oxidant. It participates in many enzymatic reactions, and in the synthesis of some proteins (collagen) and some hormones, it has a protective role against infections and detoxifying actions. Food sources of vitamin C include fruit (such as citrus fruits, strawberries, kiwi, mango, and papaya), tomatoes, peppers, cabbage, and green leafy vegetables. The main antitumor mechanisms of high-dose ascorbate include the potentiation of oxidative damage in cancer cells, the enhancement of immune function, and a decrease in inflammation, capable of triggering cell death and/or inhibiting proliferation and growth of PaC cell lines [38]. Nevertheless, its role in preventing or treating PaC remains still uncertain, and the available scientific data should be supported by more robust clinical data and phase III studies. A meta-analysis of published case–control and cohort studies reported the insufficient evidence to conclude any relationship between vitamin C intake and risk of PaC due to possible publication bias [39]. However, several clinical trials are ongoing to better elucidate the role of high-dose ascorbate treatments in combination with chemotherapy treatments in PaC patients [38].

### 2.2. Fat-Soluble Vitamins

The fat-soluble vitamins, including A, D, E, and K, have distinct biological functions and activities. They are generally found in dietary lipids and can be stored in body fats. Vitamins A and D are primarily stored in the liver, with small amounts of vitamin K, while vitamin E accumulates in adipose tissue. Excessive intake of these vitamins may lead to disorders.

PaC patients have an increased risk of fat-soluble vitamin deficiency. A typical sign of ductal pancreatic adenocarcinoma (PDAC) is ductal pancreatic insufficiency, a pathological condition resulting from the damage caused by molecules derived from carcinoma (or from surgery), which determines a malabsorptive condition of difficulty digesting or absorbing nutrients from food, thus leading to the decreased synthesis of some enzymes, including lipase, necessary for the absorption of lipids and fat-soluble vitamins. Additionally, pancreatic insufficiency due to pancreatitis (especially chronic pancreatitis) can increase the risk of developing PaC. In addition, cancer can, in turn, cause pancreatitis by inducing inflammation and/or impairing the pancreatic duct [40,41].

**Figure 2:**
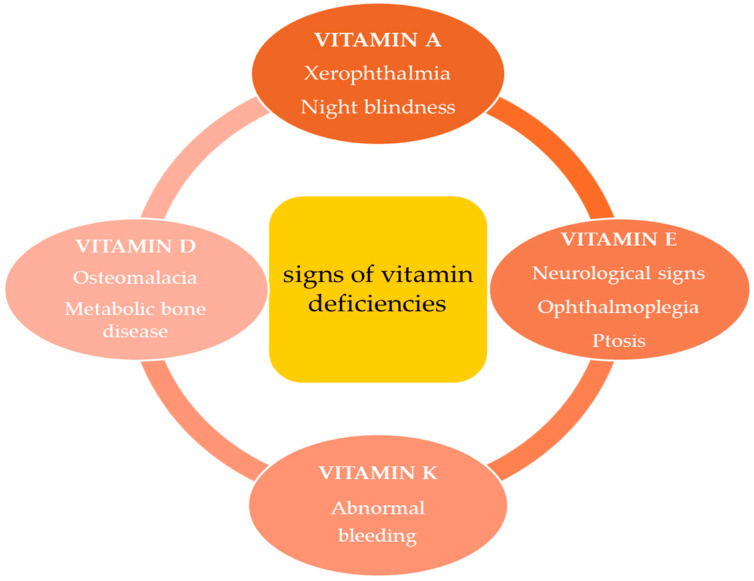
Post-pancreatectomy vitamin deficiencies due to pancreatic insufficiency.

**Figure 3:**
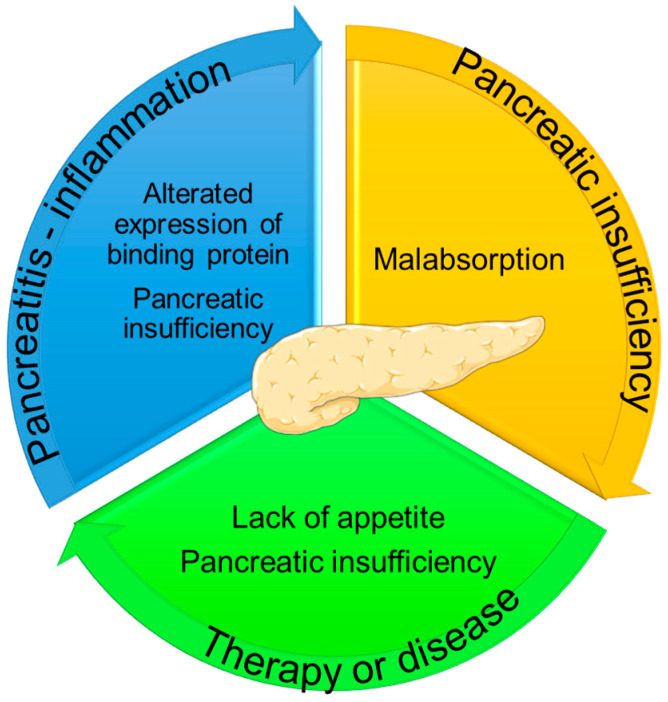
The vicious cycle of fat-soluble vitamin deficiencies in pancreatic cancer.

As shown in Figure 2, xerophthalmia and night blindness (vitamin A), neurological symptoms, ophthalmoplegia and ptosis (vitamin E), abnormal bleeding (vitamin K), and osteomalacia and metabolic bone disease (vitamin D) represent the clinical signs of specific vitamin deficiency due to pancreatic insufficiency following pancreatectomy [42]. Fat-soluble vitamins, which are involved in many processes within the body, are also known for their role in stimulating the immune system against cancer cells, and therefore, their deficiency could play a fundamental role in the intrusive pathobiology of ductal pancreatic adenocarcinoma [43]. The lack of fat-soluble vitamins and the malfunctioning of pancreatic tissue and the immune system create a vicious cycle (Figure 3).

#### 2.2.1. Vitamin A

Vitamin A is involved in vision, embryogenesis, immune function, cellular growth, and differentiation. In addition, it appears to be involved in the modulation of cellular apoptosis. The main dietary sources of vitamin A are foods of animal origin, while plant foods, especially those that are yellow or orange in color, are rich in beta-carotene, the precursor to vitamin A (provitamin A), which the body converts into the vitamin after intake. Understanding the relationship between this vitamin and cancers is complex due to several factors, like dosage, source, and individual health conditions. Retinoids derived from vitamin A are used in cancer treatment due to their role in cell differentiation and apoptosis, which can inhibit tumor growth [44]. On the other hand, excessive vitamin A intake can promote certain cancers, but its antioxidant properties protect cells from damage [45,46]. There is no clear evidence linking vitamin A levels to the onset of PaC, even though an association between lower levels of vitamin and increased risk of developing PaC has been suggested [47]. However, it is important to recognize that results can vary among studies, and establishing clear-cut differences in basal vitamin A concentrations is complex. Individual variations, dietary factors, and other variables contribute to this complexity, and understanding these factors is crucial for assessing vitamin A concentrations in PaC patients, and therefore, for evaluating and managing nutritional status in these subjects [48]. In this context, it should be taken into account that (i) poor nutrition or inadequate intake of foods rich in vitamin A can contribute to lower concentrations; (ii) PaC cancer may affect the normal functioning of the pancreas, leading to malabsorption issues, and therefore, affecting the absorption of vitamin A; (iii) vitamin A is stored in the liver, and any liver dysfunction, which can be associated with PaC, may affect its storage and release; (iv) chronic inflammation, often present in PaC, can affect vitamin A metabolism and utilization; (v) individual genetic variations can influence how the body metabolizes and utilizes vitamin A; (vi) surgery or chemotherapy may impact vitamin A levels [45]. Its deficiency can lead to reversible blindness in low-light conditions, as in the case study of a man who underwent duodenocephalopancreasectomy, chemotherapy, and radiotherapy for pancreatic adenocarcinoma and experienced deterioration of his ocular function [49]. Another study confirmed that vitamin A deficiency (and consequent blindness) may be caused by the malabsorption condition resulting from duodenocephalopancreasectomy [50]. These data are in contrast with a study reporting that no vitamin A deficiencies occurred following surgery [8]. Older studies report a lack of vitamin A in patients with PaC both associated with carcinomas in metastatic form, which then cause malabsorption [51] and pancreatic carcinoid syndrome [52]. A recent review evaluated the dysregulation of the functions of vitamin A (retinol) and its metabolites (retinoids) in PDAC and described their actions to support the development of new therapies and increase patient survival [53]. However, multiple factors, including nutritional components and individual-specific variable patterns, may play a role but are not exclusive predictors of PaC progression.

#### 2.2.2. Vitamin D

This vitamin increases the intestinal absorption of calcium and phosphorus, and it also promotes bone mineralization and immune function [54]. There are two forms of vitamin D: one found exclusively in plants (ergocalciferol, or vitamin D2) and another one that is typical of animal organisms (cholecalciferol, or vitamin D3). In addition to dietary intake, the body produces vitamin D from one of its precursors (provitamin D3) in the skin in response to sunlight. The most important dietary sources are cod liver oil, mackerel, herring, salmon, sardines, egg yolks, whole milk, and butter.

**Figure 4:**
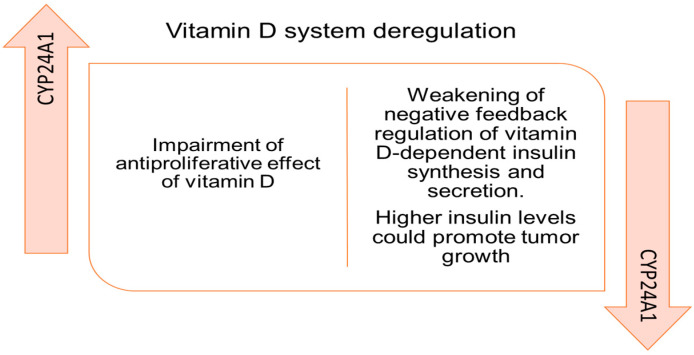
Possible vitamin D deregulation by CYP24A1 in PaC.

The relationship between vitamin D and cancer is a topic of research, and several studies have explored the potential role of vitamin D in cancer prevention and treatment [55]. Vitamin D is thought to influence cancer cells and tumor development through various pathways, including its impact on cell differentiation, apoptosis, and angiogenesis. Additionally, its immune-modulating effects may contribute to cancer prevention, and understanding the mechanisms by which vitamin D influences the immune system and the cancer process could prevent inflammation-associated cancer and improve therapeutic treatments. Inflammation due to a tumor leads to an increase in the production of C-reactive protein (CRP) and a decrease in the synthesis of vitamin D-binding protein (important for vitamin D absorption in the intestine and transport in the vessels). In this context, it should be stressed that vitamin D has a considerable role in counteracting tumor cell proliferation and that it enhances the antitumor effects of chemo- and radiotherapy; therefore, its deficiency can interfere with the survival of the subject [43,56]. Research exploring the relationship between vitamin D levels and the onset of pancreatic tumors is an active area, but the findings are not entirely consistent, and the mechanisms involved are not fully understood. Even though establishing a direct causal relationship between low vitamin D levels and the development of PaC is challenging, while some studies suggest an association, it is unclear whether low vitamin D levels contribute to the development of PaC or if they are a consequence of the disease. In addition, in PaC patients, changes in vitamin D concentrations over time can be influenced by various factors, including the progression of the disease, cancer treatments, nutritional status, and other clinical variables. Epidemiological studies have provided some evidence supporting the idea that adequate vitamin D levels may be associated with a reduced risk of PaC. However, not all studies have shown consistent results. Skinner and coworkers observed that a higher intake of vitamin D was associated with a decreased risk for PaC in two large prospective cohort studies [57]. On the contrary, an increased risk of PaC was observed with higher levels of dietary vitamin D intake in a pooled analysis from the Pancreatic Cancer Case–Control Consortium [58]. In a multicenter study drawn from a large combination of European cohort studies, estimates of the increasing effect of PaC risk with increasing pre-diagnostic vitamin D concentrations were not statistically significant [59]. A meta-analysis [60] indicated there were no significant associations between vitamin D intake or plasma 25(OH)D levels and PaC risk. Also, Shen and colleagues [61] concluded in their systematic review and meta-analysis that high plasma 25(OH)D may be associated with lower PaC mortality but not with PC incidence. Despite these encouraging results, caution in increasing vitamin D intake in the general population should be advised. Different studies have reported conflicting findings on the association between circulating basal (baseline) vitamin D concentrations and PaC, and it is crucial to note that individual variations, including geographical locations, ethnic backgrounds, demographic characteristics, and lifestyle factors can influence vitamin D levels and cancer risk. Lower baseline vitamin D levels compared to controls or reference populations are often associated with various cancers, including pancreatic. Van Loon et al. [62], in a correlative study involving 256 patients, found vitamin D deficiency (25(OH)D < 20 ng/mL) highly prevalent among patients with a new diagnosis of advanced PaC, and black patients had statistically significantly lower 25(OH)D levels than white patients (10.7 vs. 22.4 ng/mL, *p* < 0.001). The concentration of 25(OH)D has been found to be less than 30 ng/mL in 94.2% and less than 10 ng/mL in 30.1% of PaC patients [63]. Even in patients undergoing pylorus preserving duodenocephalopancreasectomy, concentrations less than 20 ng/mL in 50% of patients is comparable with the reference population [8]. However, as these patients also underwent gemcitabine-based chemotherapy, a possible correlation between the two events is plausible. In fact, cancer treatments, such as chemotherapy and radiation therapy, can impact vitamin D metabolism. Some treatments may affect the function of the liver and kidneys, which are involved in the activation of vitamin D, and side effects like nausea and fatigue may influence dietary intake, as well as sunlight exposure. In fact, PaC patients may undergo a decrease in outdoor activities and sunlight exposure due to symptoms, treatment-related side effects, or hospitalization, thus leading to a reduction in the endogenous production of vitamin D. Weight loss and malnutrition due to reduced food intake, malabsorption, and increased metabolic demands in PaC patients can lead to deficiencies in micronutrients, including vitamin D. A lower body mass index and a decrease in muscle mass are common in PaC patients. Lower BMI has been correlated with lower vitamin D levels, and individuals with higher body fat may have an altered distribution and utilization of vitamin D [64]. PaC patients may experience dysphagia (difficulty swallowing) due to the location and size of the tumor, and dysphagia can lead to decreased food intake and malnutrition, resulting in a lower absorption of fat-soluble vitamins, such as vitamin D. Additionally, pancreatic insufficiency due to cancer presence can impair fat digestion and the absorption of fat-soluble vitamins, potentially leading to lower vitamin D levels. A prospective study involving adults revealed a high incidence of pancreatic insufficiency and vitamin D deficiency in patients undergoing pancreatodenectomy over a period of 3.5 years [65]. Also, on a small group of children undergoing surgery for PaC, vitamin D was found to be insufficient (<30 ng/mL) during follow-up in 50% of patients; nonetheless, it should be stressed that low levels of vitamin D could not be exclusively related to surgical treatment [66]. Vitamin D levels in patients with GEP and NET appear to be lower than in the reference population [29], and the deficiency can worsen with the progression of the tumor stages when a condition of pancreatic insufficiency also occurs [67]. A study involving 138 patients with GEP and NET estimated that approximately 68% patients had vitamin D values < 20 ng/mL [68]. In these patients, the observed deficiency can lead to an increased risk of osteoporosis and osteopenia; this risk is further exacerbated by analogous molecules of somatostatin administered to some of these patients, causing a reduced adsorption of vitamin D taken with the diet [69]. It has also been observed that the vitamin D system is deregulated in the pancreas of PDAC patients (Figure 4). As determined by a study conducted on a small cohort of PDAC patients, an overall upregulation of both vitamin D receptors (VDRs) and the vitamin D degrading enzyme 1,25-dihydroxyvitamin-D3 24-hydroxylase (CYP24A1) has been described in tumor regions, with respect to adjacent non-tumorous tissues. However, a more in-depth analysis of CYP24A1 expression in various pancreatic cell types revealed that during tumor transformation, the expression of CYP24A1 is lost in untransformed endocrine cells, while it drastically increases in transformed ductal cells. Hence, the high CYP24A1 levels might result in greater inactivation of vitamin D, and thus, prevent the antitumor activity of this vitamin, while lower CYP24A1 levels in islets would lead to enhanced vitamin D-dependent insulin secretion and promote the growth of neighboring tumor cells in a paracrine manner [70].

The progression of PaC may also impact vitamin D concentrations. As the disease advances, changes in metabolic processes, inflammation, and overall health can contribute to fluctuations in vitamin D levels. In recent years, several studies have been conducted to evaluate whether plasma vitamin D levels could be a relevant factor for the survival of individuals with pancreatic tumors. A meta-analysis [60] indicated that the elevated plasma levels of 25(OH)D are significantly associated with improved survival in PaC patients, and recently [71], the circulating concentration of 25-hydroxyvitamin D (25(OH)D) was indicated as a potential prognostic marker in advanced PaC for positive correlation with OS. Some of these studies, carried out on patients with PaC even at an advanced stage, indicate no correlation between the plasma vitamin D levels and survival [72], neither in patients with pancreatic adenocarcinoma receiving gemcitabine-based chemotherapy [62] nor in patients with metastatic PaC also receiving gemcitabine-based chemotherapy [73]. Conversely, other studies have demonstrated that pre-diagnosis vitamin D levels are important for the prognosis of patients [74]. In adenocarcinoma, low levels of vitamin D (at diagnosis) were associated with elevated levels of inflammatory biomarkers (IL-6, chitinase-3-like protein 1, CHI3L1, and CRP) in all stages of the disease; patients with sufficient vitamin D levels in the early stages of cancer showed a longer survival than those with low vitamin D levels [75].

#### 2.2.3. Vitamin E

Vitamin E comprises a family of lipid-soluble compounds consisting of two major groups, namely tocopherols and tocotrienols. Major food sources are grains (whole grains), vegetable oil (seed and olive oil), nuts (walnut, hazelnut, and almond), liver, beef, avocado, cheese, and egg yolk. Widely recognized as a potent antioxidant, it also plays an important role as an anti-inflammatory and immune-boosting compound [76]. Vitamin E levels may be assessed by evaluating the plasma concentration of α-tocopherol and in vitro analysis of the hydrogen peroxide-treated erythrocyte lysis test.

Duodenocephalopancreasectomy can cause a significant decrease in the plasma levels of vitamin E [77], which may interfere with the efficacy of chemotherapy. In fact, vitamin E can enhance the antitumor effect of gemcitabine and decrease the nuclear factor k-dependent inflammatory pathway. This pathway is activated in 67% of PaC patients [43]. Recent evidence confirmed the role of vitamin E in modulating proliferation, cell death, angiogenesis, metastasis, and inflammation in PaC cells [78], although the precise effects on these cells are still to be fully clarified.

#### 2.2.4. Vitamin K

Vitamin K is essential for the body to synthesize clotting factors, special proteins able to stop bleeding when a blood vessel is injured. Its deficiency is very rare in adults because a balanced diet is sufficient to meet its requirement. Food sources comprise green leafy vegetables (spinach and lettuce), Brassicaceae (broccoli and cabbage), cereals, meats, and dairy products. There is not available evidence through clinical trials to fully support the role of this vitamin in PaC intervention, although pre-clinical studies demonstrate vitamin K antiproliferative activity through the activation of apoptosis and the inhibition of cellular growth [79], therefore, suggesting its role as a single agent or in combination with chemotherapy for treatment post-resection. In fact, following pancreatoduodenectomy, Kroon et al. [65] highlighted that pancreatic exocrine insufficiency led to vitamin K deficiency during postoperative follow-up, indicating malabsorption.

## 3. Conclusions

Although the available literature is limited, it can be inferred that in PaC patients, the vitamins most at risk of deficiency are thiamine (B1), niacin (B3), pyridoxine (B6), and certain fat-soluble vitamins. Cancer patient management necessitates the consideration of their nutritional status to prevent symptoms associated with such deficiencies. The literature data concerning vitamins, such as cobalamin (B12), vitamin C, and vitamin K, are scarce, incomplete, or conflicting. One explanation for the lack of studies evaluating vitamins in PaC patients could be that some vitamins are rarely determined in clinical routine.

In summary, several key points regarding the role of vitamin malnutrition in the development and progression of PaC should be considered. Chronic deficiencies of certain vitamins, often associated with a poor diet lacking in fruits, vegetables, and other nutrient-rich foods, can heighten vitamin deficiencies and, consequently, the risk of PaC. Vitamins are essential for a healthy immune system and a weakened immune system may struggle to detect and destroy cancer cells, potentially facilitating cancer development. Furthermore, certain vitamins have anti-inflammatory properties, and since chronic inflammation is considered a risk factor for PaC, deficiencies in anti-inflammatory vitamins may exacerbate this risk. Also following a healthy lifestyle, including abstaining from smoking, helps preserve essential vitamins and antioxidants that aid in protecting against PaC.

Unfortunately, the existing literature data predominantly comprise case studies or studies with small sample sizes of individuals, which inevitably poses limitations; therefore, future studies should aim at confirming the existence of these vitamin deficiencies and elucidating their impact on patient survival and quality of life.

## 4. Materials and Methods

A bibliographic search was carried out in PubMed and checked by examining SCOPUS, Embase, and Web of Science. Studies published in the last 10 years on humans were considered. The keywords used were the common and chemical names of each vitamin, followed by “status and pancreatic cancer” or “assessment and pancreatic cancer” or “deficiency and pancreatic cancer”. The retrieved articles included reviews and case–control and case studies that reported a significant correlation between PaC patients and vitamin and/or nutritional deficiencies. Our search was limited to reports in English language; to identify additional publications, we also extended our search to the articles included in the reference lists of the retrieved studies.
